# Impact of water–rock interaction on the pore structures of red-bed soft rock

**DOI:** 10.1038/s41598-021-86815-w

**Published:** 2021-04-01

**Authors:** Meiling Zhou, Jianlin Li, Zuosen Luo, Jianbin Sun, Feng Xu, Qiao Jiang, Huafeng Deng

**Affiliations:** 1 Key Laboratory of Geological Hazards on Three Gorges Reservoir Area of Ministry of Education, China Three Gorges University, Yichang, 443002 Hubei China; 2 College of Hydraulic and Environmental Engineering, China Three Gorges University, Yichang, 443002 Hubei China; 3 China International Water and Electric Co., Ltd., Beijing, 100032 China; 4 China Three Gorges Projects Development Co., Ltd., Chengdu, 610041 Sichuan China

**Keywords:** Civil engineering, Petrology

## Abstract

The physical and mechanical properties of the reservoir bank slope are affected by the water–rock interaction. However, few studies considered the impact of long-term water–rock interaction on the evolution law of mesostructure. Therefore, in this study, the water–rock interaction test was conducted on a slightly weathered red-bed soft rock from the Three Gorges Reservoir area, considering the fluctuation in the reservoir water level. The corresponding pore structure parameters were measured and analyzed based on a scanning electron microscope (SEM) and digital image processing technology. The study showed that: (1) The pore size has been gradually increased, while the number of pores was increased initially and then decreased. Within 12 cycles, the maximum and average pore radius of the rock specimens was increased by 101.02% and 43.32%, respectively, and the porosity has been increased by 26.59%, whereas the number of pores decreased by 22.65%. This indicates the effect of water–rock interaction on the propagation of pores. (2) The pores were changed from oblate to slender by the water–rock interaction. The shape factor was decreased by about 15.79% within 12 cycles. In the meantime, the fractal dimension was increased from 1.20 to 1.28, and more complex structures of pores were observed. (3) The porosity evolution model for the red-bed soft rock was established based on the curve fitting technique. The results can be used as a reference to conceptualize the mesostructure damage of rocks under water–rock interaction.

## Introduction

The reservoir bank slopes have been affected by the long-term water–rock interaction. The cyclic impact of the reservoir water (scouring, dissolution, and erosion) alters the microstructure, physical and mechanical characteristics of the rock mass, and the stability of the bank slope^1–3^. In particular, slopes composed of mudstone, limestone, and other soft rocks are significantly affected by the water–rock interaction^4,5^.

The impacts of water–rock interaction on the physical and mechanical properties variation of rock have been well studied in the past. Erguler et al.^6–10^ respectively analyzed and summarized the weakening effect of water and chemical solution on the strength, stiffness, and solid substrate of different kinds of rocks. Valès et al.^11–14^ further studied the deterioration degree of mechanical strength and physical properties of rock with different water content, and illustrated the transformation of their failure mode. Moreover, considering the engineering condition, Hale et al.^15–21^ designed different dry–wet cycling tests to simulate the water–rock interaction, and established the degradation mechanism and failure characteristics of different rocks based on experimental investigations. The related researches on mesoscale and microscale were gradually carried out, too.

Previous studies have shown that the water–rock interaction developed and changed the structure of rock^22–25^. Liu et al.^26,27^ and Liu et al.^5^ investigated the microstructure of sandstone and granite rocks under cyclic dry–wet conditions. The findings revealed that the water–rock interaction significantly altered the composition and structural characteristics of the rocks. Sausse et al.^28^ and Zhuang et al.^29^ analyzed the crack pattern of granite under water–rock interaction with different water content. The results showed that the water–rock interaction propagated the crack pattern. Song et al.^30^, Deng et al.^31,32^ examined SEM and core thin-section images of limestone, sandstone, and red-bed soft rock. Accordingly, the water–rock interaction yielded concentration and propagation of micropores and cracks.

The above findings laid a solid foundation for the degradation mechanism of rock by the water–rock interaction. Besides, the water–rock interaction significantly alters the mesostructure of rocks in many ways. However, the impact of long-term water–rock interaction on the mesostructure characteristics of rocks is seldom studied statistically.

Therefore, in this paper, a slightly weathered red-bed soft rock was taken from the Three Gorges Reservoir area as the research object. Besides, water–rock interaction tests were carried out to capture the fluctuation in the reservoir water level. Finally, the pore structure parameters were quantitatively analyzed based on SEM and digital image processing technology, to establish evolution law for the rock pore structure under long-term water–rock interaction.

## Material and methods

### Sample preparation

**Figure 1:**
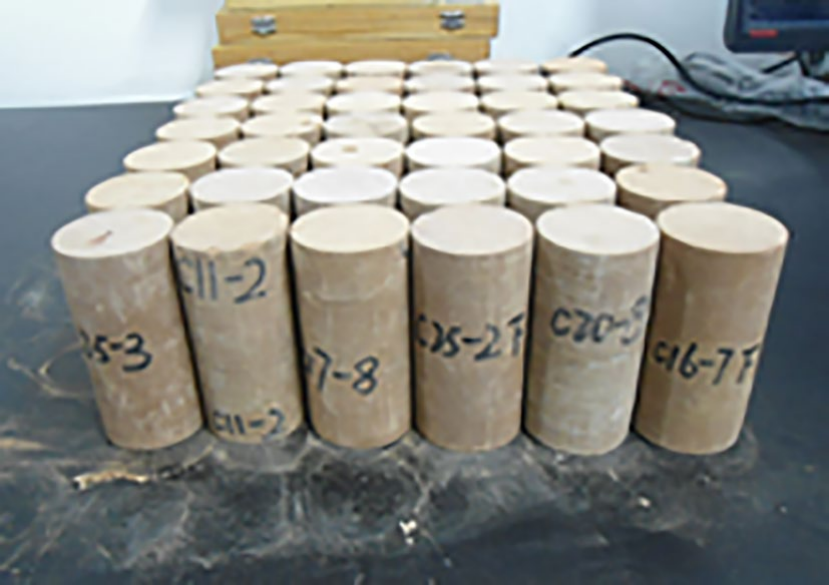
Prepared rock samples.

The red-bed soft rock used in the experiment was taken from the reservoir water fluctuation zone of a typical reservoir bank slope in the Three Gorges Reservoir area. Which belongs to the Triassic Badong Formation and is reddish-brown argillaceous siltstone with a fine texture. And it is mainly composed of quartz, feldspar, calcite, dolomite, and some clay minerals. The rock samples (shown in Fig. 1) are drilled from blocks with good unity and obvious layers, according to the Rock Test Rules for Water Conservancy and Hydropower Engineering (DLT5368-2007), and the International Society for Rock Mechanics (ISRM).

The weight, density, and P-wave velocity of the prepared samples were measured, and the samples with larger dispersions were kicked out^33^ in case the inner structural defects affecting the test results.

### Water–rock interaction simulation

**Figure 2:**
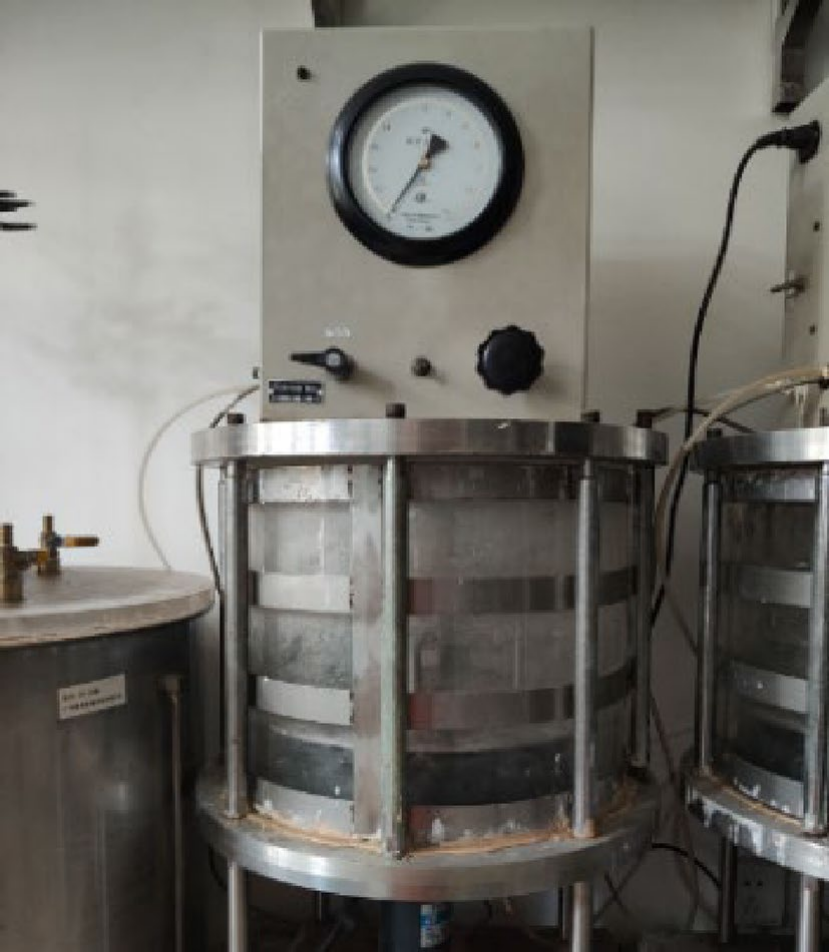
YRK-1 rock dissolution apparatus.

The dissolution apparatus is shown in Fig. 2, which was invented by the research group to simulate the water–rock interaction. Three floors in the apparatus can hold 60 samples in total and the water pressure in the apparatus can be adjusted from 0.05 to 1.2 MPa. So that the fluctuation of water pressure in China Three Gorges Reservoir Area can be well simulated with this device. Furthermore, the water for immersion was taken from the Yangtze River near the Sampling sites. According to the water pressure variation in the studied area, the immersion pressure was set to 0–0.3 MPa.

**Figure 3:**
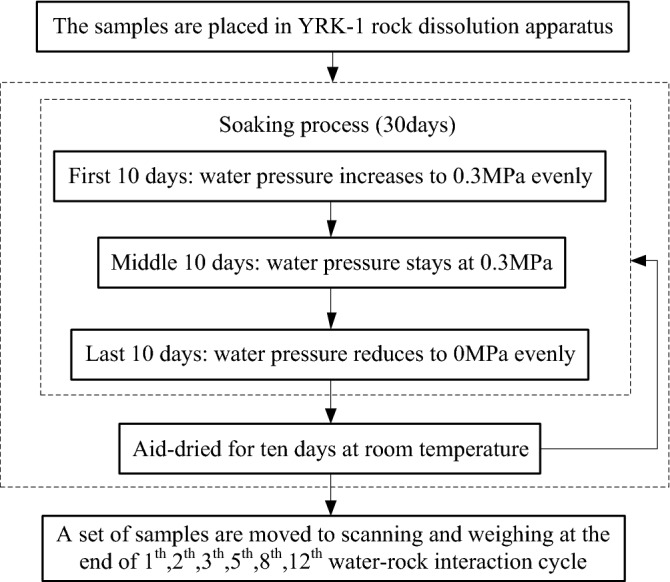
Flow chart of water–rock interaction test.

Rock samples were numbered from R1 to R12, according to the water–rock interaction cycles they suffered. And the flow chart of water–rock interaction is shown in Fig. 3. Each water–rock interaction cycle lasting for 40 days, 30 days for soaking, and 10 days for air-drying. During the soaking, the water pressure increased uniformly from 0 to 0.3 MPa at the first 10 days, to simulate the process of the reservoir water rise in the storage period; and then remained at 0.3 MPa during the middle 10 days, to simulate the high-water level period; in the last 10 days, water pressure decreased uniformly from 0.3 to 0 MPa, to simulate the drop of reservoir water level in flood discharge period. And then, the samples were air-dried for 10 days at room temperature, to simulate the natural air-dry process of the bank slope at a low water level.

### Test porosity calculation

The dry weight of the samples was measured before the water–rock interaction test, while the floating weight and saturated weight of the samples were measured in every water–rock interaction cycle. Finally, the porosity (*n'*, named test porosity) of rock samples is calculated according to Eq. (1).

1 $${\mathrm{n}}^{\mathrm{^{\prime}}}=\frac{{V}_{\mathrm{a}}}{{V}_{\mathrm{a}}{+V}_{\mathrm{b}}}=\frac{({m}_{2}-{m}_{0})/{\rho }_{w}}{({m}_{2}-{m}_{0})/{\rho }_{w}+({m}_{2}-{m}_{1})/{\rho }_{\mathrm{w}}}=\frac{{m}_{2}-{m}_{0}}{2{m}_{2}-{m}_{0}-{m}_{1}}$$

where *V*_*a*_ is the volume of pores, *V*_*b*_ is the volume of rock, *m*_*0*_ is the dry weight of the sample, *m*_*1*_ is the floating weight of the sample, and *m*_*2*_ is the saturated weight of the sample, *ρ*_*w*_ is the density of water.

The weights and test porosity of rock samples are shown in Table 1.

**Table 1:** Weights and test porosity of rock samples.

Sample No.	Dry weight, m_0_/g	Floating weight, m_1_/g	Saturated weight, m_2_/g	Test porosity, n’/%
R1	445.10	283.86	477.45	14.32
R2	440.26	278.63	477.55	15.79
R3	439.36	277.13	480.97	16.95
R5	442.60	278.49	487.98	17.81
R8	446.73	283.25	494.77	18.51
R12	445.89	281.02	495.65	18.82

### SEM slice preparation

By the end of each water–rock interaction cycle, taking two samples to make SEM slices, one was a test sample and the other was a spare sample, the spare sample was used when the test one failed to be split or sanded.

From each rock sample, three slices were cut to be scanned and analyzed. And the average value of the three slices was specified as the value of the sample, to reduce accidental errors.

**Figure 4:**
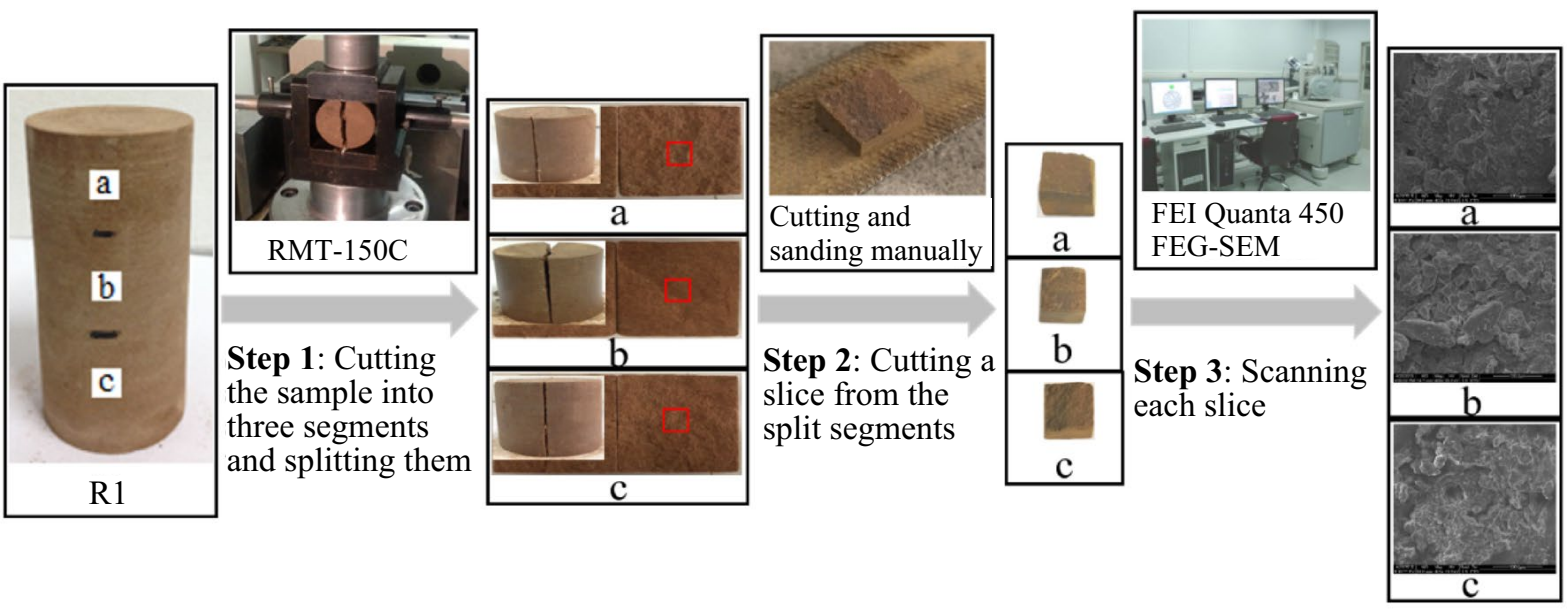
Production and scanning of SEM slice.

Sample R1 is taken as an example to illustrate the production and scanning process of SEM slices. The specific steps are shown in Fig. 4. Firstly, cutting the sample into three segments. Numbering the segments a, b, and c, respectively, and splitting them by RMT-150C rock mechanics test system. Secondly, cutting the segments into SEM slices. Cutting a slice from the split segment, with the length of each side within 10 mm. The split surface of the segments was retained as the upper surface to be scanned and the other five surfaces of the slice were cut and sanded manually to protect the upper surface from damage. Finally, using FEI Quanta 450 FEG-SEM field emission scanning electron microscope to scan the slices and get their SEM images. Similarly, the processes were applied to samples R2–R12.

### Computer identification of the pore morphology

According to previous studies^34,35^, Images with a magnification of 400 times were selected for the analysis of the pore structure, since the SEM images of too large magnification may lose most of the pores, and too small magnification may miss the small holes.

The SEM images were segmented by the Otsu method, considering the segmentation principle of the Otsu method and the gray distribution characteristics of the image, the grayscale interception method was applied to make the image gray more equalizer before the segmentation to eliminate the effect of uneven image exposure on pore recognition. Moreover, the isolated points in the segmented images were settled by open and closed operation and removed according to the area of each independent block.

**Figure 5:**
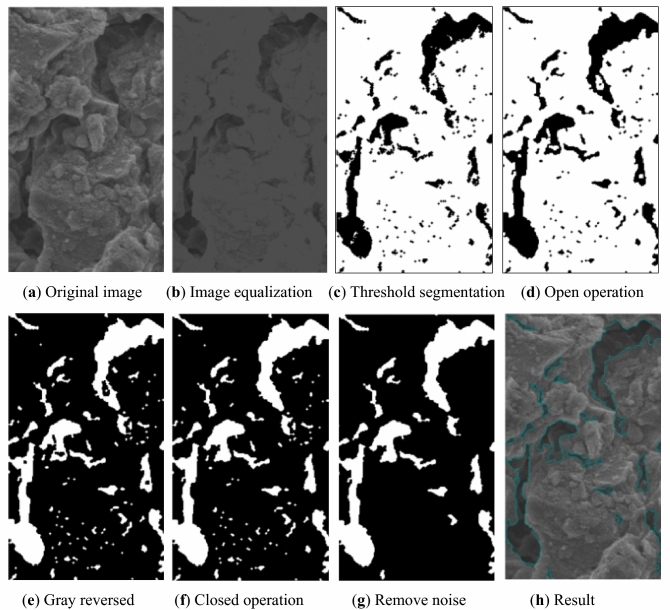
Computer identification steps.

Based on the classification of pores (shown in Table 2) by Zhang et al.^36^, only gas can penetrate in pores with a radius smaller than 1 μm. Since the study was designed to evaluate the impact of water–rock interaction on the pore structures, this study just considers pores with a radius equal to and greater than 1 μm, including mesopores, macropores, and super macropores. The area of a pixel on the 400 times magnified image is approximately 0.108 μm^2^, the number of pixels covered by a mesopore with a radius of 1 μm is about 29. Therefore, blocks less than 29 pixels were eliminated as noise. Specifically, the steps of image processing are shown in Fig. 5.

**Table 2:** Classification of pore and the number of pixels corresponding.

Category	Radius, μm	Area, μm^2^	Pixels number	Characteristic
Ultra micropore	< 0.01	< 0.000314	< 1	Gas adsorption zone
Micropore	0.01–0.1	0.0003–0.0314	< 1	Gas condensation and diffusion zone
Small pore	0.10–1.0	0.0314–3.1400	1–29	Gas slow laminar flow and penetration zone
Mesopore	1.00–10	3.1400–314.00	29–2907	High-pressure liquid seepage zone
Macropore	10.0–100	314.00–31,400	2907–290,741	Natural water seepage zone
Super macropore	> 100	> 31,400	> 290,741	Natural water seepage zone

Once the individual pores are identified, the number of the pores, the area, the radius, and the perimeter of each pore in different water–rock interaction cycles can be collected. Furthermore, the evolution law of the radius, shape factor, orientation, and fractal dimension of the pores by water–rock interaction can be calculated and analyzed.

## Results and discussion

### Effect of water–rock interaction on the number and radius of pores

Pore radius is an important parameter to characterize the microstructure of porous media and has an essential effect on the permeability and mechanical properties of rock. Due to the extremely irregular shape and the random distribution in size, it is not easy to determine the rock pore radius according to a particular direction or scale. Therefore, the equivalent radius was applied to represent the radius, and the pore area *S*_*a*_ was considered as a circular area. Accordingly, the equivalent radius *R* is:

2 $$R=\sqrt{\frac{{S}_{a}}{\pi }}$$

**Figure 6:**
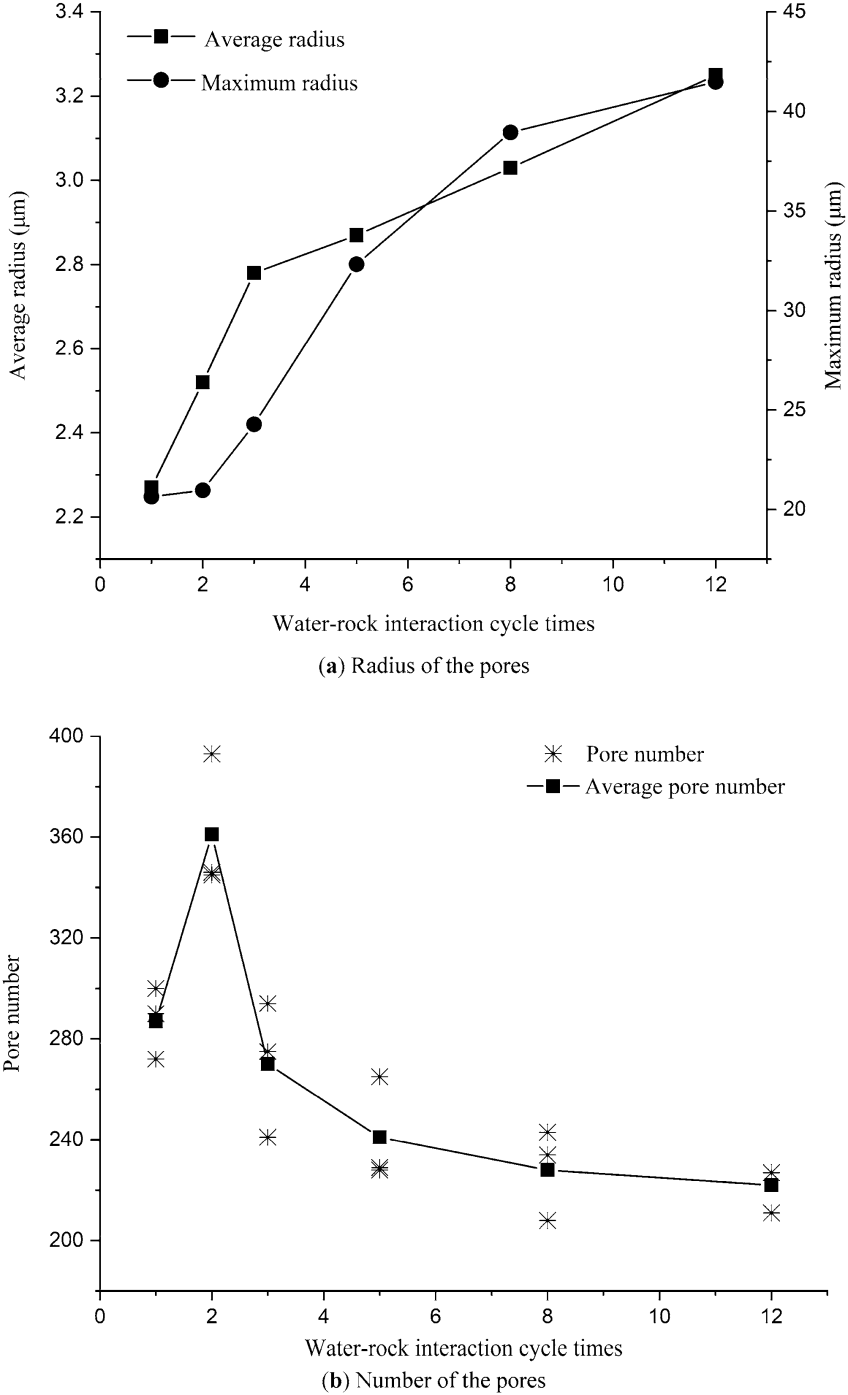
Radius and number variation rules of the pores by water–rock interaction.

The numbers of pores and their radius are shown in Table 3. And Fig. 6 shows the variation of pore number and pore radius in water–rock interaction.

**Table 3:** Pore number and radius of red-bed soft rock in different water–rock interaction cycles.

Sample no.	Images no.	Average radius $\stackrel{-}{r} (\mathrm{\mu m})$	Mean $\stackrel{-}{R} (\mathrm{\mu m})$	Maximum radius ${\mathrm{R}}_{max}(\mathrm{\mu m})$	Mean $\overline{{{\mathrm{R}}_{max}}}(\mathrm{\mu m})$	Pore number	Mean
R1	a	2.53	2.27	21.43	20.64	290	287
	b	2.07		21.12		300	
	c	2.20		19.38		272	
R2	a	2.39	2.52	19.76	20.96	393	361
	b	2.52		22.74		346	
	c	2.65		20.39		345	
R3	a	2.87	2.78	22.68	24.27	241	270
	b	2.62		25.88		294	
	c	2.84		24.26		275	
R5	a	2.80	2.87	32.07	32.33	228	241
	b	2.87		31.74		229	
	c	2.93		33.17		265	
R8	a	3.32	3.03	34.82	38.95	234	228
	b	2.84		42.30		243	
	c	2.94		39.75		208	
R12	a	3.17	3.25	41.00	41.49	211	222
	b	3.48		40.75		227	
	c	3.09		42.73		227

It can be seen from Fig. 6a that both the average pore radius and maximum pore radius increased gradually. The average radius grew rapidly in the first 3 cycles and increased by 43.32% within 12 cycles. The maximum pore radius hardly developed in the first two cycles but rose sharply during the third to eighth cycles, eventually, the maximum pore radius increased by 101.02% within 12 cycles. Figure 6b illustrates that the number of pores increased initially and then decreased. It increased by 25.75% in the second cycle and then reduced continuously. After 12 cycles, the number of pores diminished by 22.65%.

According to the rock pore size distribution proportion by Zhang et al.^36^, the micro and small pores account for about 45% of the volume of pores in natural rock. Thus at the early stage of water–rock interaction, a large number of micro and small pores extended or combined into mesopores. Although some of the mesopores transformed into macropores at the same time, the number of newly added mesopores is much larger than the number of mesopores that transformed into macropores. As a result, the number of pores increased by 25.75% at the end of the second cycle. Three water–rock interaction cycles later, most of the easily connected micro and small pores have completed their interconnection process. Since then, the changes in pores mainly depended on the extension and combination of mesopores and macropores. Therefore, the number of pores gradually decreased, and the increase rate of the average radius also slowed down 3 cycles later.

Water–rock interaction cycles will dissolve and shed the minerals at the edges of the pores. Which on the one hand will increase the area of the pores, on the other hand, will connect the adjacent pores and form them into a larger one. In the meantime, some of the closed pores are connected to open pores. All these activities will make the pores expand and connect, and eventually increase the pore size and reduce the pore number.

### Effect of water–rock interaction on the shape factor of pores

The shape factor is used to characterize the roundness of the pore boundary. Through comparing the parameters and methods commonly used in morphological analysis, Tu and Wang^37^ pointed out that the parameter *T* can well describe the particle shape. Referring to this, this paper employed *T* to describe the shape of the pores. It is defined as:

3 $$T=\frac{2\sqrt{\pi {S}_{a}}}{C}$$

where *T* is the shape factor of the pores, *C* is the perimeter of the pores, and *S*_*a*_ is the areas of the pores. The perimeter *C* is the sum of the outer lengths of the pixels at the pore boundary.

The shape factor and typical pore morphology of red-bed soft rock in each water–rock interaction cycle are presented in Table 4.

**Table 4:**
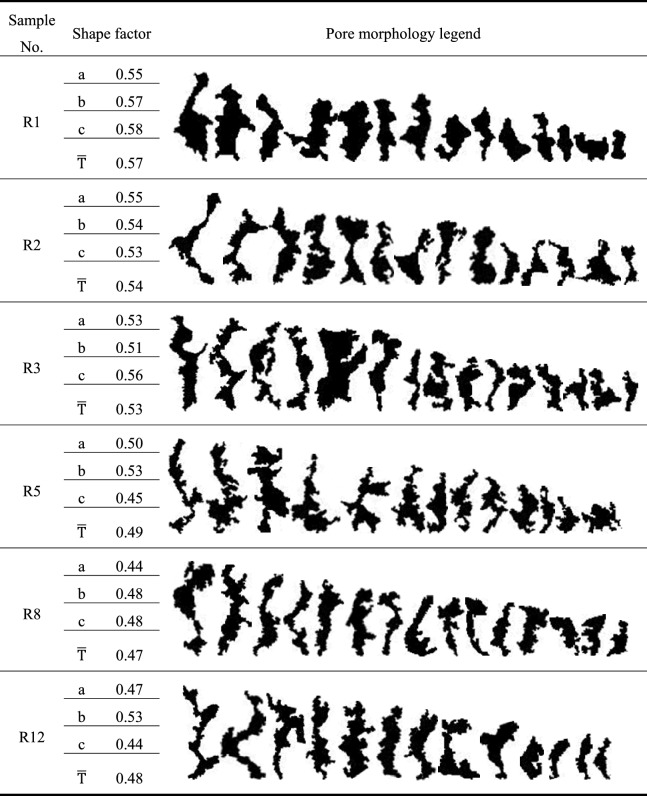
Pore shape factor of red-bed soft rock in different water–rock interaction cycles.

**Figure 7:**
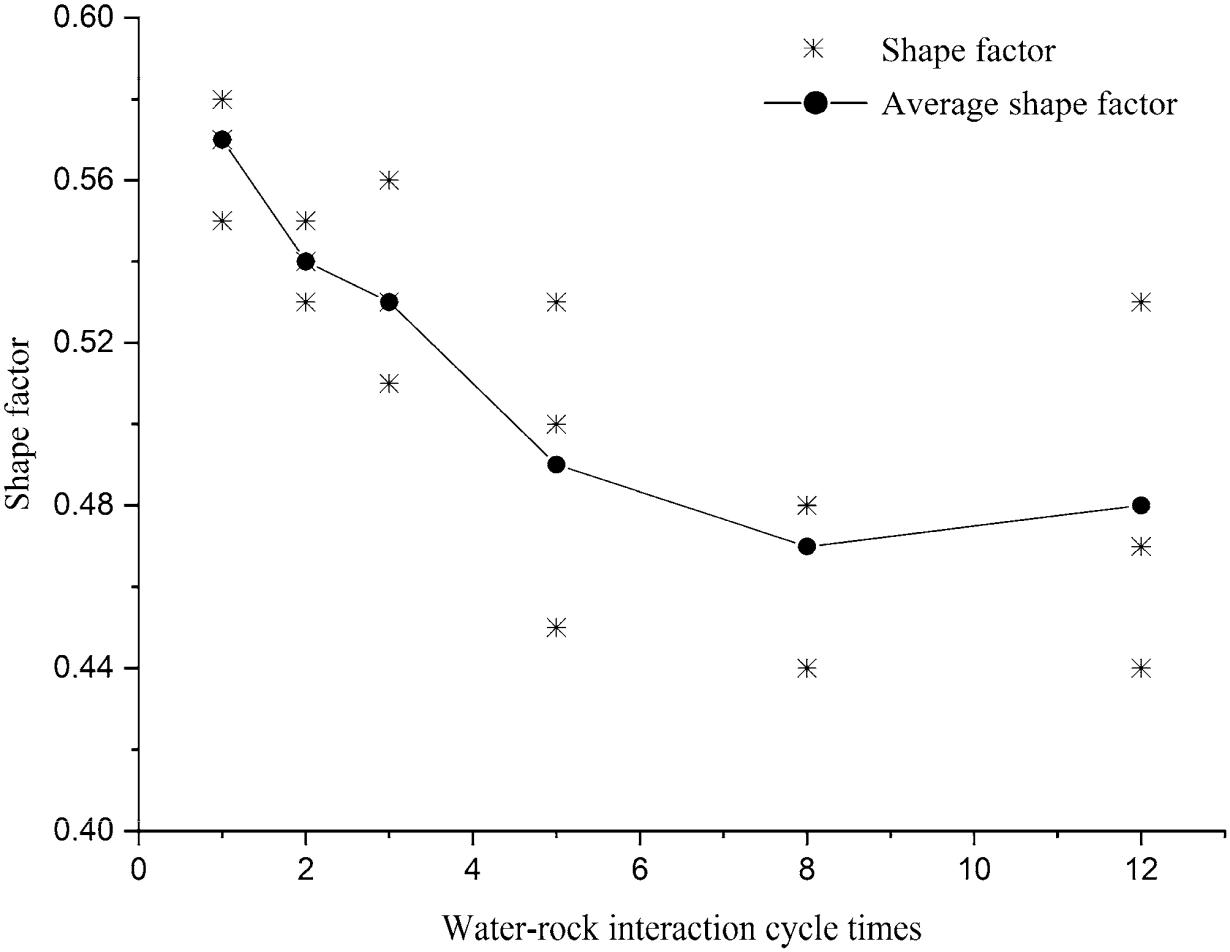
Shape factor variation rule of the pores by water–rock interaction.

According to Eq. (3), the values of the pore shape factors range from 0 to 1. The shape factor of a circle, a regular octagon, hexagon, and quadrilateral are 1, 0.974, 0.952, and 0.886, respectively. Therefore, the pore shape is rounder when the *T* is closer to 1. In contrast, a smaller *T* means a more irregular pore shape. Moreover, there will be prominent sharp angles on the pore boundary when *T* is less than 0.886. The pore shape factor evolution curve of red-bed soft rock samples by water–rock interaction is exhibited in Fig. 7.

It can be seen from Table 4 and Fig. 7 that the shape of the pores became more complicated as the number of water–rock interaction cycles increased. The shape factor of the pores decreased from 0.57 to 0.48, declined by about 15.79% within 12 water–rock interaction cycles. The morphology legends of typical pores in different water–rock interaction cycles showed that the convex structures at the edge of the pore structure were progressing, and the shape of the pores gradually changed from oblate to slender. To conclude, water–rock interactions dissolved and shed minerals at the edges of the pores, making the original pores extend. In the meantime, the access of some micropores made the pore edges slender. As a result, the area of the pores increased, and the shape of the pores grow complicated.

### Effect of water–rock interaction on the orientation of pores

Generally, the rock pores have a definite direction along the structural surface. For contours with smooth curves, two parallel lines tangent to the contour curve are usually used to determine the direction of the pores: changing the position of the parallel lines continuously to get the long axes of the pores, and the direction of the long axis is the direction of the pores. However, the pores analyzed in this study are contoured by an orderly, square border, so the parallel tangent lines are inapplicable. Computers can solve this problem with their powerful data processing capabilities, to get an accurate value, the ergodic method was used to find the long axes of the pores. Traversing all the points on the edge of the pore, and finding out the two with the longest distance, the line between them is the long axes of the pore, and the direction of the line is the direction of the pore. When there are more than one long axes, taking the vector sum of the long axes as the pore direction.

Shi^38^ cited the probability entropy to reflect the arrangement order of microstructure units in clay. Similarly, this article cited the probability entropy to reflect the orientation of the pore structure in rocks. The probability entropy *H* is defined as:

4 $$H=-\sum_{i=1}^{n}{P}_{i}{\cdot \mathit{log}}_{n}{P}_{i}$$

where ${P}_{i}$ is the frequency of the pores in the direction within range *i*.

The range of pore direction is 0°–180°, setting every 10° as a unit, *n* = 18. For example, when *i* = 1, ${P}_{i}$ is the frequency of the pores with the direction ranges from 0° to 10°.

The value of probability entropy *H* ranges from 0 to 1. *H* = 0 means all the pores are in the same direction. Whereas *H* = 1 means the pore directions are randomly distributed, in other words, the probabilities of pores in all directions are the same. So, a smaller *H* value means a more unanimous pore direction, and a larger *H* value means a more random pore direction. The probability entropy of red layer-soft rock in different water–rock interaction cycles are shown in Table 5.

**Table 5:**
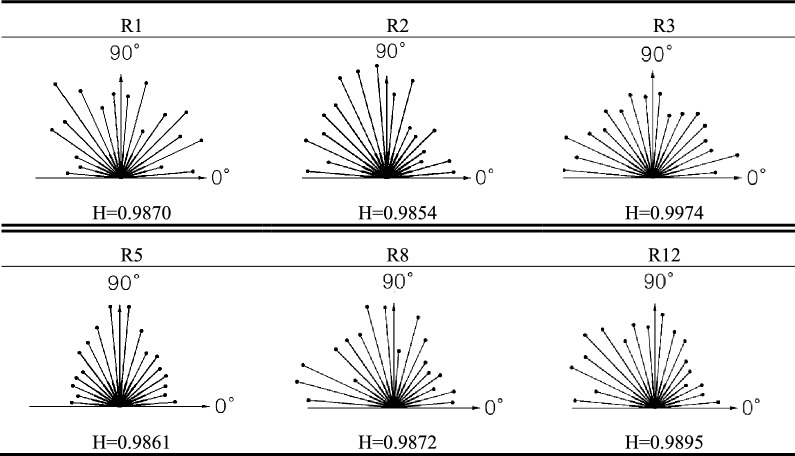
Probability entropy of red-bed soft rock in different water–rock interaction cycles.

It can be seen from Table 5 that the probability entropy values of all the samples were higher than 0.98, indicating that the distribution of pores in all directions was close. Besides, as the number of water–rock interaction cycles increased, the probability entropies haven't show a regular change of increase or decrease, but slightly fluctuated between 0.985 and 0.998. It revealed that in cyclic water–rock interaction, the expansion and connection of pores inside the samples altered the direction of the pores, thus caused the probability entropies of the pores to change. But the overall distribution of pore orientations was hardly changed, so the probability entropies of all the samples were greater than 0.98. On the whole, it illustrates that the distributions of pores in all directions within the rock are always balanced in periodic water–rock interaction.

### Effect of water–rock interaction on the fractal dimension of pores

Rock pores have remarkable fractal features according to the existing researches^39,40^, and the perimeter-area relationship is customarily applied to figure the fractal dimension of closed curves. For a regular pattern, perimeter *C* is proportional to the measurement size *λ*, and the area *S* is proportional to *λ*^*2*^, which can be expressed as:

5 $$\mathrm{C}\propto {S}^{1/2}$$

However, for irregular patterns, Mandelbort^41^ proposed to replace the smooth perimeter in Eq. (5) with a fractal perimeter curve, thus gained the following relationship:

6 $${[\mathrm{C}\left(\uplambda \right)]}^{1/D}=a{\uplambda }^{\left(1-D\right)/D}{[S(\uplambda )]}^{1/2}$$

where *D* is the fractal dimension, *a* is a constant related to the shape of the pattern, and* λ* is the measurement size.

Equation (6) will transform into Eq. (5) when *D* = 1. The length of a pixel in this paper is set to 1. Plotting log{[S(λ)]^1/2^/λ} ~ log[C(λ)/λ] scatter diagram of the pores, and fitting the points with a line, the slope of the line is the fractal dimension of the pattern.

**Figure 8:**
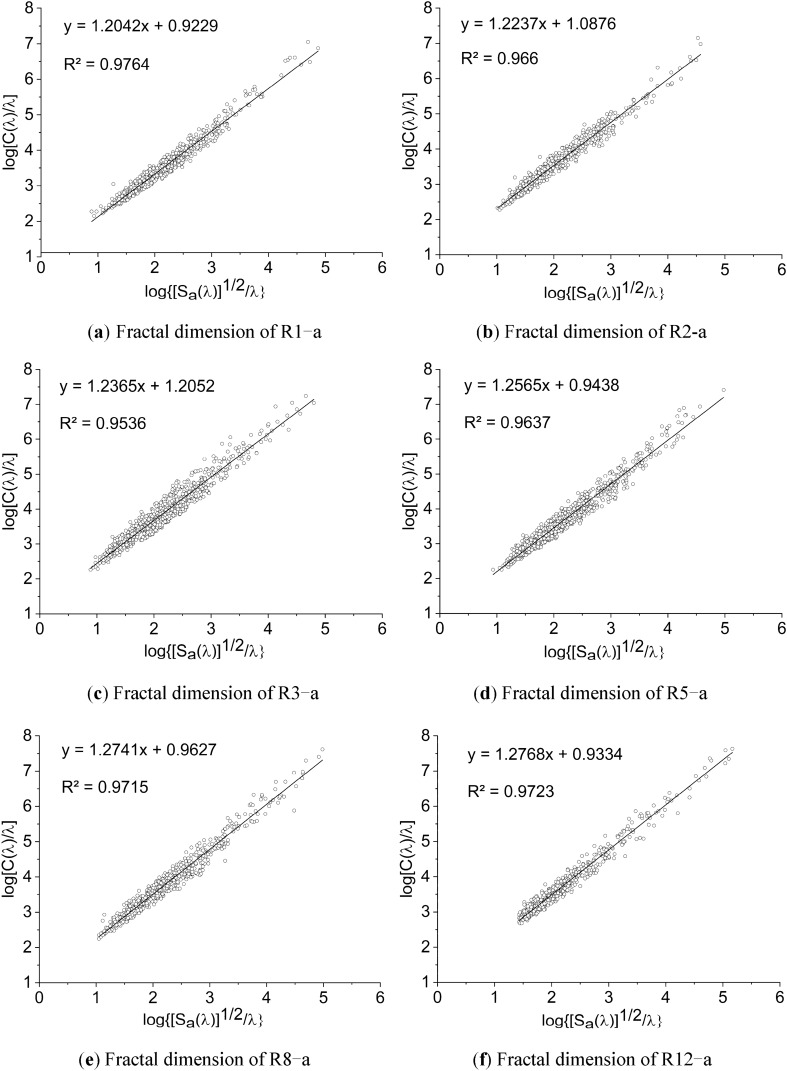
Fractal dimension fitting graphs of the pores in different water–rock interaction cycles.

The fractal dimensions of different samples are shown in Table 6, and the representative fitting formulas and fitting graphs of the samples are presented in Fig. 8.

**Table 6:** Fractal dimension of red-bed soft rock in different water–rock interaction cycles.

No	R1	R2	R3	R5	R8	R12
a	1.2042	1.2237	1.2365	1.2565	1.2741	1.2768
b	1.2222	1.2548	1.2601	1.2416	1.3126	1.2870
c	1.2287	1.2347	1.2416	1.2899	1.2629	1.2965
Mean	1.2184	1.2377	1.2461	1.2627	1.2832	1.2868

**Figure 9:**
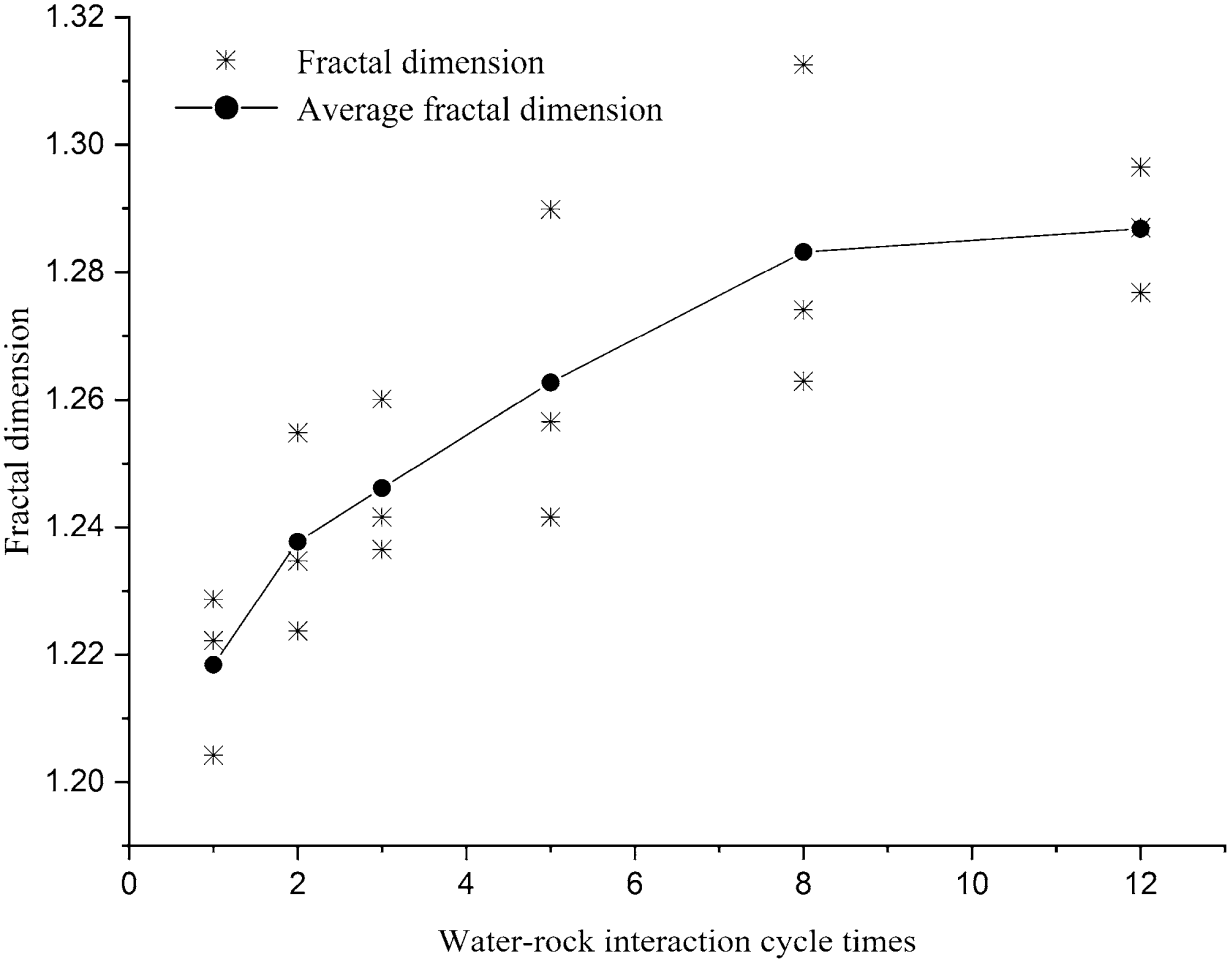
Fractal dimension variation rule of the pores by water–rock interaction.

It can be seen from the fitting graphs that the scatter points are regularly distributed on the perimeter-area logarithmic coordinate system, and the linear fitting correlation coefficients of the points are higher than 95%. These indicate that the pores of red-bed soft rock are self-similar, so the fractal dimension can typify the fractal characteristics of the pores in red-bed soft rocks well. The pore fractal dimension curve of different samples are shown in Fig. 9.

The fractal dimension of the pores of red-bed soft rocks altered from 1.20 to 1.28 as the water–rock interaction cycles increased, up by about 6.67% within 12 cycles. The increase of fractal dimension implied that the water–rock interaction made the pore structures more complex. That is consistent with the analysis of the pore shape factor. Furthermore, according to the research of Cheng et al.^42^, rock strength will decrease with the increase of pore fractal dimension. Since the fractal dimension of red-bed soft rock increased by water–rock interaction, the strength of red-bed soft rock would decrease gradually on this condition.

### Effect of water–rock interaction on porosity

The porosity was calculated according to the area of the pores. As stated earlier, computer identification can distinguish the pores and substrate in the SEM images, and the pore area *S*_*a*_ and the substrate area *S*_*b*_ can be calculated according to the number of pixels they contain. The porosity (*n*, named digital porosity) can be expressed as:

7 $$n=\frac{{S}_{a}}{{S}_{a}{+S}_{b}}\times 100\%$$

**Figure 10:**
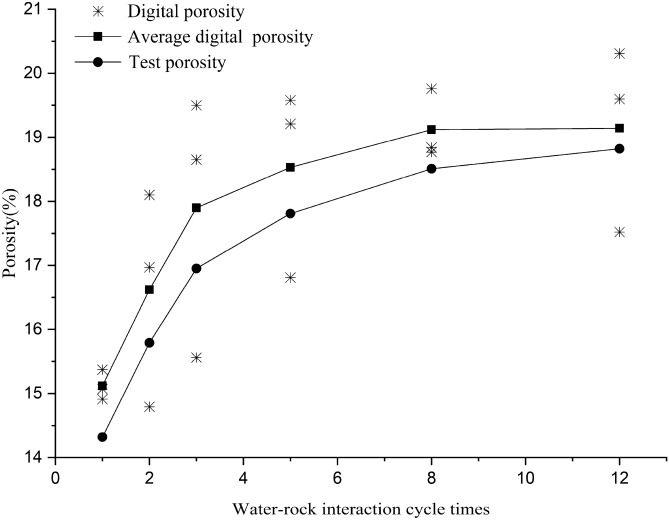
Porosity variation rule of the pores by water–rock interaction.

The porosities of red-bed soft rock samples in different water–rock interaction cycles are shown in Table 7. The porosity variation law is shown in Fig. 10.

**Table 7:** Porosity of red-bed soft rock in different water–rock interaction cycles.

Sample no.	Images no.	Digital porosity. $\mathrm{n}$ (%)	Average digital porosity, $\stackrel{-}{\mathrm{n}}$ (%)	Test porosity, ${\mathrm{n}}^{\mathrm{^{\prime}}}$(%)	Error, (%)
R1	a	14.91	15.12	14.32	5.56
	b	15.37			
	c	15.07			
R2	a	16.97	16.62	15.79	5.26
	b	14.79			
	c	18.1			
R3	a	19.5	17.90	16.95	5.62
	b	18.65			
	c	15.56			
R5	a	16.81	18.53	17.81	4.06
	b	19.58			
	c	19.21			
R8	a	18.84	19.12	18.51	3.31
	b	18.77			
	c	19.76			
R12	a	17.52	19.14	18.82	1.72
	b	20.31			
	c	19.60

It can be seen from Table 7 that the digital porosities of red-bed soft rock are close to the test ones, since both the open pores and the closed pores are all included in the SEM images, the digital porosity is slightly higher than the test porosity. That is because as the number of water–rock interaction cycles increases, some closed pores gradually connect with the open ones, the pores counted by the test method and digital method are getting closer, therefore the values of digital porosity and the test porosity are approaching. Figure 10 shows a similar pattern between the digital porosity and the test porosity, suggesting that the pores extraction method in this study is reliable.

The porosity of red-bed soft rock samples increased with the water–rock interaction cycles. In the first five cycles, the porosity increased rapidly, the test porosity and digital porosity increased by 24.37% and 22.55% respectively. After 5 cycles, the increase slowed down, the increase of the test porosity and digital porosity from the 6th to 12th cycles was 7.05% and 4.03% respectively, which is much less than that in the first five cycles. Indicating that with the increase of water–rock interaction cycles, the porosity of red-bed soft rock will gradually stabilize.

**Figure 11:**
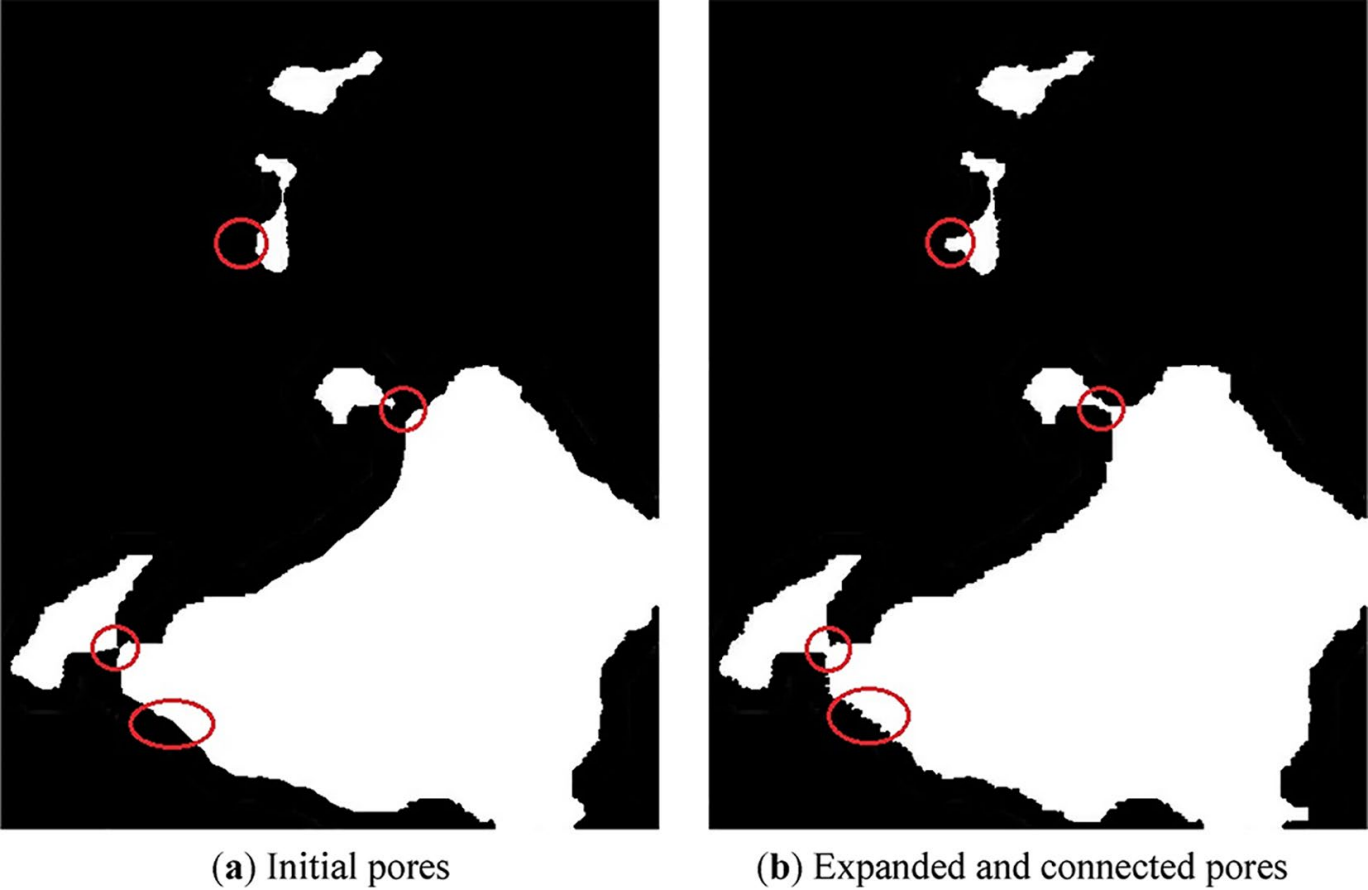
Expanding and connecting of the pores.

In the natural state, the structure and stress distribution in the rock is uniform. However, under the water–rock interaction cycle, a variety of physical, chemical, and mechanical activities will occur inside the rock, such as the expansion and contraction of clay minerals during immersion and air drying, the dissolution and ion exchange of water-soluble minerals, the storage and release of pore water pressure^30^. As analyzed in the previous analysis, these effects will lead to the expansion and connection of the pores, as shown in Fig. 11, which eventually led to an increase in rock porosity and rock deterioration.

**Figure 12:**
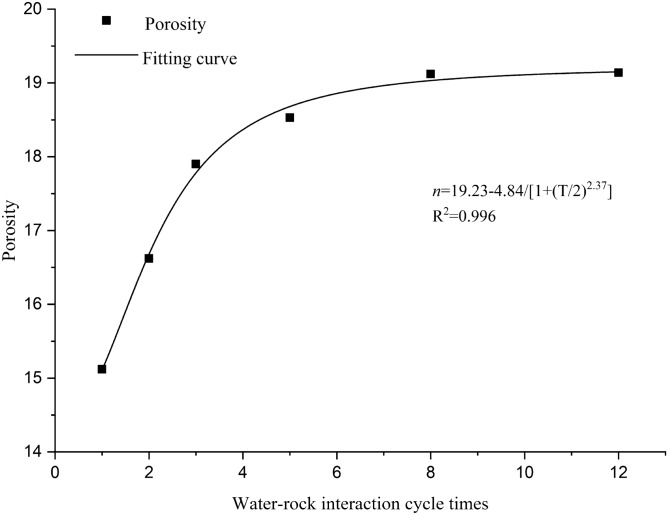
Porosity fitting curve of red-bed soft rock.

According to the porosity variation law of the red-bed soft rock in water–rock interaction, the logistic linear regression function is applied to fit the porosity. The fitting formula is shown in Eq. (8), and the fitting curve is shown in Fig. 12.

8 $$n = {19}.{23} - {4}.{84}/\left[ {{1} + \left( {T/{2}} \right)^{{{2}.{37}}} } \right]$$

where *n* is the porosity of the rock, and *T* is the water–rock interaction cycle times.

The fitting curve has a high correlation with the digital porosity. The results can be used as a reference to conceptualize the mesostructure damage of rocks under water–rock interaction.

## Conclusions

In this paper, the pore structure evolution characteristics of red-bed soft rock in periodic water–rock interaction were studied combined with the water–rock interaction test and SEM digital image processing technology. The main conclusions are as following:

The pore size of the red-bed soft rock gradually increased, while the number of pores increased initially and then decreased. Within 12 water–rock interaction cycles, the maximum pore size and average pore size of the samples increased by 101.02% and 43.32%, respectively. The number of pores increased by 25.75% in the second cycle and then decreased continuously, and dropped by 22.65% after 12 cycles.

The shape of the pores was changed from oblate to slender by the water–rock interaction, and the pore structure became more complicated. As water–rock interaction cycles increased, the shape factor of the pores gradually decreased, and the fractal dimension gradually grew. Within 12 cycles, the shape factor decreased by 15.79%, and the fractal dimension increased by 6.67%.

Water–rock interaction changed the direction of the pores, but in general, the distributions of pores in all directions within the rock are always balanced in water–rock interaction, thus the probability entropy of pores fluctuated slightly between 0.985 and 0.998.

The porosity of the red-bed soft rock gradually increased in water–rock interaction cycles, and the first 5 cycles contributed most of the growth. Specifically, the porosity of the samples increased by 22.55% within the first 5 cycles, and by 26.59% in total. Moreover, the porosity evolution model for the red-bed soft rock was established based on the curve fitting technique, and the fitting results matched the measured one well.

The deterioration of rock mesostructure is mainly reflected in the variation of shape and size of the pores. On one hand, water–rock interaction increased the slender structure at the edge of the pores and made the pore shape more complex. On the other hand, water–rock interaction made the tiny pores connect to the larger ones, and increased the pore size and porosity of the rocks.

## Data Availability

The software MATLAB was used to process and calculate the images in this study, and no custom code was used in this study.

## Data availability

The authors confirm that the data supporting the findings of this study are available within the article.
